# Exercise and Ischemia-Activated Pathways in Limb Muscle Angiogenesis and Vascular Regeneration

**DOI:** 10.14797/mdcvj.1304

**Published:** 2023-11-16

**Authors:** Vihang A. Narkar

**Affiliations:** 1 Brown Foundation Institute of Molecular Medicine, McGovern Medical School, UTHealth, Houston, Texas, US

**Keywords:** exercise, angiogenesis, limb ischemia, peripheral artery disease

## Abstract

Exercise has a profound effect on cardiovascular disease, particularly through vascular remodeling and regeneration. Peripheral artery disease (PAD) is one such cardiovascular condition that benefits from regular exercise or rehabilitative physical therapy in terms of slowing the progression of disease and delaying amputations. Various rodent pre-clinical studies using models of PAD and exercise have shed light on molecular pathways of vascular regeneration. Here, I review key exercise-activated signaling pathways (nuclear receptors, kinases, and hypoxia inducible factors) in the skeletal muscle that drive paracrine regenerative angiogenesis. The rationale for highlighting the skeletal muscle is that it is the largest organ recruited during exercise. During exercise, skeletal muscle releases several myokines, including angiogenic factors and cytokines that drive tissue vascular regeneration via activation of endothelial cells, as well as by recruiting immune and endothelial progenitor cells. Some of these core exercise-activated pathways can be extrapolated to vascular regeneration in other organs. I also highlight future areas of exercise research (including metabolomics, single cell transcriptomics, and extracellular vesicle biology) to advance our understanding of how exercise induces vascular regeneration at the molecular level, and propose the idea of “exercise-mimicking” therapeutics for vascular recovery.

## Introduction

Exercise has fantastic health benefits, with positive effects on metabolic and cardiovascular fitness that promote wellness and delay myriad conditions such as diabetes, obesity, heart failure, stroke, and peripheral arterial disease, as well as neurodegenerative diseases and dementia.^1,2,3^ While exercise can directly impact multiple organs and cell types, at the heart of the aforementioned conditions is endothelial dysfunction and impaired vascular regeneration leading to perfusion inefficiency, hypoxia, ischemic injury, metabolic dysfunction, and tissue degeneration. A prime example of these collective pathologies is peripheral artery disease (PAD) originating from vascular insufficiency in the limb musculature.^4,5^ Currently, there are no effective pharmaceutical treatments for PAD (or other cardiomyopathic diseases) to promote vascular regeneration in ischemic tissue.^6,7^ The only partially effective options include endovascular surgery or management of underlying causes such as hypertension, atherosclerosis, obesity, and diabetes.^6,7,8^

Notably, regular exercise mitigates cardiovascular complications such as PAD, and rehabilitative physical activity is effective in enhancing vascular function.^9^ Exercise can directly impact vasculature either through vascular remodeling or improving endothelial function, as well as by promoting vascular regeneration by vasculogenesis, arteriogenesis, and angiogenesis—overall, resulting in improved tissue perfusion and aerobic capacity. Unfortunately, conditions such as obesity, diabetes, aging, and progressive cardiovascular diseases impair mobility and ability to exercise, further worsening vascular dysfunction. In this scenario, in-depth understanding of the mechanisms of exercise-induced vascular health could facilitate “exercise-mimicking” therapeutics for vascular regeneration.

Here I review key pathways through which exercise might promote angiogenesis and vascular regeneration. I have focused on the mechanism of limb muscle angiogenesis in exercise, ischemia, and PAD. This focus is justified as skeletal muscle is a major organ recruited during exercise that undergoes vascularization in response to physical activity. Therefore, it is an excellent model system to investigate the interaction between exercise and vascular regeneration. Further, I identify future areas of research that will help advance exercise therapy or development of exercise-mimetic chemical approaches for vascular regeneration and cardiovascular diseases.

## PAD and Exercise Intervention

PAD originates from occlusion of large blood vessels and may be further associated with microvasculature regression, which leads to decreased blood flow and ischemic damage to the limb musculature.^6,10^ In the past decade, the incidence of PAD has jumped by over 21%, with approximately 200 million people worldwide suffering from this cardiovascular complication.^7^ Over 12 million cases of PAD were reported in 2015 in the United States.^11,12^ Atherosclerosis is the most frequent cause of PAD, and accordingly factors that increase the progression of atherosclerosis, such as smoking, diabetes, obesity, hypertension, and aging, increase the risk of PAD.^12^ Clinically, PAD may be asymptomatic, or present with atypical symptoms. The classic symptomatology of intermittent claudication is apparent in only 10% to 30% of patients. The most severe cases present with critical limb ischemia, when patients present with foot pain at rest, muscle wasting, non-healing foot ulcers, and/or gangrene.^13^

The treatment of risk factors (smoking cessation, glucose control, regulation of lipid and blood pressure levels) and thrombotic risk can reduce mortality and limb loss in PAD,^14^ yet pharmacological treatments are only modestly effective for relieving the symptoms of PAD. However, clinical evidence has emerged showing that exercise therapy is a highly effective strategy for mitigating PAD symptoms and delaying disease progression. Both aerobic and strength exercise, but particularly aerobic exercise (eg, walking, minor-to-moderate treadmill exercise), is clinically effective in relieving pain, improving 6-minute walking time and distance, and improving ankle-brachial index (measure of PAD severity).^9,15,16,17^ The clinical benefits of exercise are thought to be broadly associated with lowering inflammation, reversing endothelial dysfunction, improving vascular tone, and increasing the oxidative and mitochondrial capacity of the ischemic limb musculature.^9^ In addition, exercise may reduce ischemia, and restore muscle mass and its function by promoting limb angiogenesis and vascular regeneration.

## Exercise-mediated Vascular Regeneration: A Key

Preclinical studies of muscle angiogenesis in exercise and ischemia provide valuable insights into potential signaling mechanisms of vascular regeneration by exercise in clinical settings. Highlighting angiogenesis as a key mechanism of limb muscle vascularization, its regulation by exercise or ischemia involves orchestration between muscle and muscle stem cells, immune cells, and endothelial progenitor cells to provide angiogenic growth and other factors for vascular regeneration.^18,19,20^ In addition to angiogenesis, exercise can stimulate vasculogenesis, arteriogenesis, and collateral vessel formation potentially involving angiogenic growth factors and/or activation of nitric oxide synthesis in endothelial cells.

While exercise can have a direct effect on multiple cell types in the skeletal muscle, major impacts of training are on mitochondrial function, as reviewed elsewhere,^21^ as well as through molecular activation of contracting skeletal muscle, imparting both local myocellular effects, and paracrine effects on activating non-muscle cell types such as endothelial cells to promote vascular regeneration. Key myocellular signaling pathways that may drive vascular regeneration by exercise in paracrine fashion are discussed below, followed by a brief description of other cell types through which exercise may mediate limb vascularization.

### Paracrine Angiogenesis by Muscle-derived Factors

Skeletal muscle is a reservoir of angiogenic factors (eg, vascular endothelial growth factor A, angiopoietin 1, fibroblast growth factor 1), which are upregulated and released in the skeletal muscle milieu in response to exercise and ischemia.^22,23^ In addition to pro-angiogenic factors, skeletal muscle also expresses and secretes anti-angiogenic factors.^22,23^ The balance of pro- versus anti-angiogenic factors leads to optimized activation of endothelial cells and angiogenesis to stimulate capillary formation and perfusion. It is likely that these factors also are involved in expansion of other cell types such as pericytes and smooth muscle cells (SMC) that are involved in blood vessel formation.

As mentioned, in terms of vascular regeneration, individual exogenous angiogenic factors are inefficient.^6,11^ However, key exercise-activated tissue-specific signaling pathways that orchestrate angiogenic factor gene programs could be better therapeutic targets for promoting regenerative angiogenesis. Recent studies using pharmacology or genetic mouse models have shed light on the potential pathways through which skeletal muscle-derived angiogenic factors may promote limb vascularization. Central to this mechanism is a group of transcriptional factors particularly belonging to nuclear receptor super-family, transcriptional cofactors, as well as kinases that have emerged as master-regulators of paracrine angiogenesis in the skeletal muscle in both exercise and ischemia. Representative central regulators are highlighted below.

#### Nuclear Receptors

Nuclear receptors are hormone-activated transcriptional factors that have evolved structurally to express unique ligand binding pockets.^24,25,26,27^ Several members of the nuclear receptor family are involved in exercise adaptation and ischemia-induced limb muscle vascularization. Well-characterized among them are the estrogen-related receptors (ERR): ERRa and ERRg. These receptors induce mRNA and protein expression of vascular endothelial growth factor A (VEGFA) in muscle cells, which can activate endothelial cells and promote angiogenesis in paracrine fashion.^28,29^

Transgenic mouse lines with skeletal muscle-specific overexpression of either ERRa or ERRg exhibit increased baseline vascularization as well as enhanced ischemic neo-angiogenesis in response to murine hindlimb ischemia.^30,31^ Mitigative effects of ERRs on vascular regression and ischemic neo-angiogenesis also have been demonstrated in diabetic mice, where metabolic dyshomeostasis is associated with vascular dysfunction and impaired angiogenesis.^32,33^ Transcriptionally, ERRs induce a comprehensive angiogenic program—similar to exercise training—in the skeletal muscle that facilitates neoangiogenesis and vascular regeneration via paracrine secretion of angiogenic factors.^30,31^ Recently, muscle-specific ERRa and ERRg double knockout mice were generated^34^ that exhibit poor exercise tolerance and vascular regression in the skeletal muscle, further underscoring the role of endogenous muscle ERRa/g signaling in angiogenesis. Notably, ERRs are induced by exercise and therefore might be involved in exercise-mediated adaption in the skeletal muscle.^35,36,37^ Indeed, ERRa knockout mice fail to undergo exercise-induced muscle vascularization.^38^

Whether loss of endogenous ERRs in the skeletal muscle hampers revascularization in murine hindlimb ischemia remains to be examined. Notably, expression of ERRs are repressed in diabetic skeletal muscle, which correlates with aberrant ischemic limb vascularization in a diet-induced murine obesity model.^33^ Nevertheless, gain-of-function studies using gene transfer have demonstrated that targeting ERRs is a viable therapeutic approach for mimicking exercise for vascular regeneration in PAD.^39^

Multiple peroxisome proliferator activator receptor (PPAR) agonists (eg, cilostazol, GW0742, pioglitazone, pemafibrate, and fenofibrate) stimulate ischemic angiogenesis and/or prevent capillary regression in rodent models of hindlimb ischemia and diabetes.^40,41,42,43,44,45,46^ These agents induce angiogenic stimulators including VEGFA and endothelial nitric oxide synthase (eNOS) in the ischemic muscle tissue. Notably, PPARd agonist GW1516 behaves as an exercise mimetic, improving exercise capacity in mice.^47^ Interestingly, endothelial and endothelial progenitor cells also express PPARs, where these receptors regulate angiogenesis, and thus may be involved in vascular regeneration through stem cell recruitment.^48,49,50^ New studies using muscle-specific PPAR transgenic mice^51,52,53^ are necessary to understand the precise molecular mechanisms involving PPAR agonist effects in the skeletal muscle, and further whether muscle-specific overexpression or loss of PPARs modulate exercise and ischemia-induced angiogenesis. It is noteworthy that modulation of PPARd expression through transgenic overexpression or knockout in the skeletal muscle is associated with increase or decrease in exercise tolerance,^52,53,54^ respectively, underscoring the possibility that PPARd-mediated muscle angiogenesis, in particular, could be part of training adaptation.

Classic steroid hormones through their receptors can promote skeletal muscle angiogenesis and limb vascularization. For example, testosterone promotes ischemic vascular regeneration in the murine limb in an androgen receptor (AR) dependent manner.^55^ Testosterone-mediated angiogenesis involves activation of hypoxia inducible factor 1a (HIF1a) in the ischemic muscle and bone marrow derived progenitors. Notably, loss of AR in the skeletal muscles of the mice decreases baseline capillarity in AR knockout mice and impairs revascularization in hindlimb ischemia.^56^ Androgens may function in ischemic revascularization by recruiting bone marrow-derived progenitor cells to the site of ischemia and stimulation of vasculogenesis.^57^ Apart from androgens, estrogen or estrogen mimetic agents also promote ischemic angiogenesis in the skeletal muscle. In ovariectomized female mice or rabbits, estrogen deficiency impairs ischemic revascularization,^58,59,60^ whereas compounds with estrogenic properties, such as Aucubin, promote revascularization in estrogen receptor b (ERβ) dependent fashion.^61^ The effects of estrogen may involve activation of AKT, eNOS, and VEGFA in the skeletal muscle.

Despite the robust effects of steroids on muscle angiogenesis, the precise contribution of skeletal muscle AR and ER to steroid-induced ischemic revascularization remains to be defined through use of conditional knockout mice. The molecular and transcriptional mechanisms through which muscle steroid receptors control paracrine angiogenesis beyond activation of VEGFA and eNOS also requires further study.

#### Nuclear Receptor Coactivators

Nuclear receptors orchestrate their transcriptional program by interacting with coactivators and corepressors. A key cofactor that drives exercise angiogenesis and revascularization is peroxisome proliferator activator receptor-gamma coactivator alpha (PGC1a). PGC1a is induced by exercise, hypoxia, and ischemia in the skeletal muscle.^28^ Muscle-specific overexpression studies have demonstrated that PGC1a drives the expression of VEGFA to promote ischemic muscle angiogenesis.^28^ Further, muscle PGC1a promotes macrophage recruitment in SPP1 dependent fashion,^62^ which contributes to muscle angiogenesis. PGC1a is also involved in exercise-induced angiogenesis.^38^ PGC1a-dependent angiogenesis is shown to be dependent on activation of ERRa in the skeletal muscle, which in turn drives angiogenic factor expression.^28^ Additionally, truncated isoforms of PGC1a, namely NT-PGC1a and PGC-1a4, also drive VEGFA expression and angiogenesis in the skeletal muscle.^63^

Interestingly, another coactivator belonging to the same family, PGC1b, promotes angiostatic gene expression in the skeletal muscle and endothelial cells and is involved in suppressing ischemic muscle angiogenesis.^64^ The angiostatic transcriptional effects of PGC1b involve activation of orphan nuclear receptor COUP-II.^64^ Whether PGC1b modulates exercise-induced angiogenesis is unclear. However, it should be noted that a contrasting study assigns a pro-angiogenic role for PGC1b in the skeletal muscle.^29^ While there are other major nuclear receptor transcriptional coregulators (eg, NCoRs) expressed in the skeletal muscle,^65^ their role in exercise-induced angiogenesis remains to be explored.

#### Hypoxia-inducible Factors and Related Factors

Hypoxia-inducible transcriptional factors (HIF) include HIF1a, HIF2a, HIF1b, and AHR (aryl hydrocarbon receptor) receptors. HIF1b is an obligatory heterodimerizing partner for HIF1a, HIF2a, and AHR. HIF1a is a hypoxia-inducible factor via prolyl hydroxylases. Stabilization of HIF1a expression by chemical^66,67^ or genetic^68^ inhibition of prolyl hydroxylases leads to muscle angiogenesis and revascularization in ischemia. Likewise, phosphodiesterase 5 (PDE-5) inhibitor mediated muscle angiogenesis involves stabilization of HIF1a and VEGFA induction.^69^ Skeletal muscle gene transfer of HIF1a or HIF2a also leads to angiogenesis, providing direct evidence for HIF involvement in muscle vascularization.^70^ However, muscle or embryonic muscle-specific HIF1a and HIF2a double knockout mice have normal muscle development and mass and seem to be dispensable for muscle vascularization at baseline or in exercise.^71,72^ Likewise, muscle-specific deletion of HIF1b (an obligate binding partner of HIF1a/2a) also does not affect muscle angiogenesis and vascularization.^73^

Aryl hydrocarbon receptor (AHR) also interacts with HIF1b but generally has opposing effects to HIF1a and HIF2a. AHR expression is increased in skeletal muscle in association with chronic kidney disease and murine hindlimb ischemia or PAD, and muscle-specific deletion of AHR improves muscle reperfusion, mass, and function.^74^ Notably, endothelial-specific AHR overexpression impairs ischemic angiogenesis in murine hindlimb ischemia.^75^ Therefore, AHR is a negative regulator of vascular regeneration. The potential impact of muscle AHR expression on exercise-induced muscle angiogenesis and vascularization needs to be further explored, but overexpression of AHR in the skeletal muscle induces muscle wasting akin to inactivity or smoking–lifestyle choices that impair angiogenesis and vascular function.^76^

Interestingly, various reports point to an interaction between the HIF1a and ERRa (described above). At least under hypoxic conditions, induction of ERRa in muscle cells is HIF1a dependent, and hypoxia mimetic agents such as CoCl2 and DMOG can induce ERRa expression.^31^ Notably, ERRg does not seem to interact with hypoxic signaling in the skeletal muscle.^36^ HIF1a has been shown to physically interact with ERRs to drive transcription, albeit in non-muscle cells.^77,78^ Thus, while there is no direct report of HIF1 being involved in exercise-mediated angiogenesis, it is possible that it may interact with ERRs in driving paracrine angiogenesis (and metabolic adaptations) in both exercise and ischemia.

#### AMP Activated Protein Kinase

AMP activated protein kinase (AMPK) is a stress activated protein kinase and a master-regulator of skeletal muscle homeostasis.^79^ Global AMPK α1 and α2 knockout mice have impaired ischemia-induced angiogenesis and reperfusion after femoral vessel ligation.^80^ Muscle-specific AMPK dominant negative mutant transgenic mouse studies suggest that AMPK is required for basal muscle vascularization but is dispensable for exercise-induced angiogenesis.^81^ Other studies with pharmacological activators such as metformin or AICAR also demonstrate that muscle AMPK activation is associated with improved neoangiogenesis and ischemic revascularization.^82,83^

Notably, AICAR is also able to improve exercise tolerance in mice, in part via skeletal muscle remodeling.^47^ Interestingly, AMPK activation induces ERRa and recruitment of the receptor to angiogenic gene promoters.^33^ Accordingly, AMPK-dependent angiogenic gene induction depends on ERRa in muscle cells.^33^ AMPK also has been shown to interact with the PPARs,^47^ and therefore its paracrine angiogenesis effects might involve more than one nuclear receptor.

**Figure 1:**
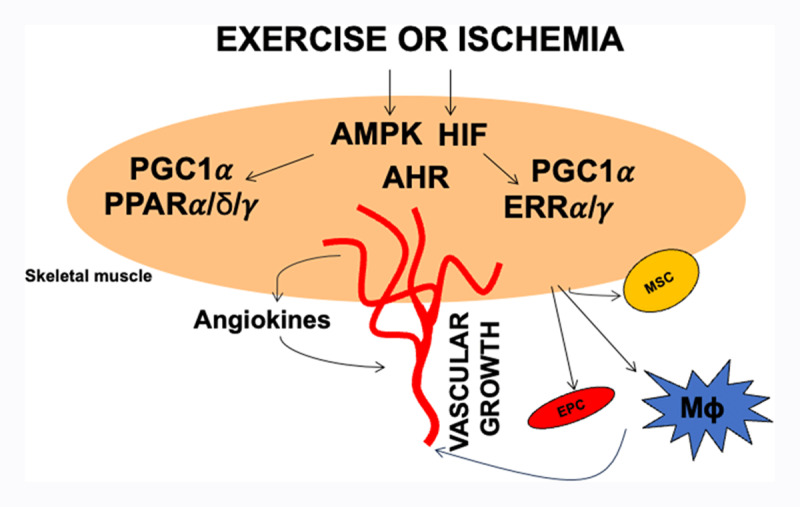
Exercise and ischemia activated paracrine angiogenesis program in skeletal muscle. Exercise and ischemia activate multiple signaling pathways in the skeletal muscle including AMP-activated protein kinase (AMPK), hypoxia inducible factors (HIF), and transcriptional regulators such as peroxisome proliferator activated receptors (PPARs), estrogen-related receptors and peroxisome proliferator activated receptor co-activator 1 alpha (PGC1α). These factors drive paracrine angiogenesis programs that promote vascular regeneration. They may also drive secretion of other myokines that can activate or recruit muscle stem cells, endothelial progenitor cells, or immune cells such as macrophages to promote vascular regeneration.

To summarize, several myocellular transcriptional regulators have emerged that respond to exercise and/or ischemia, or can be simply elicited by synthetic ligands to promote muscle vascularization. These regulators include nuclear receptors and their coregulators and HIF family members expressed in the skeletal muscle. In addition, kinases such as AMPK might act as sensors of exercise activity and signal to various downstream transcriptional factors to drive angiogenic gene programs (Figure 1). Multiple other signaling pathways are involved in skeletal muscle adaptation to exercise,^84,85^ which might be potentially involved in muscle angiogenesis, although their description is beyond the scope of this review. Furthermore, several of the aforementioned molecular pathways also are expressed in the endothelial and other vascular cells^86,87^ and therefore could mediate the effect of exercise directly on vascular cells, particularly via blood flow changes and shear stress during physical activity.

## Other Mechanisms of Exercise-induced Vascular Regeneration

Additional mechanisms could play a role in exercise or ischemia-induced vascular regeneration in the limb muscles. As stated, vascular regeneration in response to exercise may involve (1) immune cells releasing cytokines and angiogenic growth factors, (2) muscle stem cells acting as a depot of angiokines or hypothetically transdifferentiating into angiogenic cells, and (3) endothelial progenitor cells migrating to the vasculature of limb muscles.^19,20,88,89,90^ Exercise or ischemia can activate and/or recruit the aforementioned cell types in the limb muscle. However, exercise-linked signaling pathways that orchestrate these different cell types remain to be elucidated in detail. Furthermore, exercise or ischemia-activated myokines may play a major role in recruiting these cells to the site of vascular regeneration.^91^ These additional mechanisms are summarized in Figure 1.

## Summary and Future Directions

Vascular regeneration in cardiovascular conditions such as PAD has remained a formidable challenge. Exercise has long been known to have preventive or rehabilitative effects in cardiovascular diseases. In PAD, regular exercise can improve pathological parameters, improve mobility, and delay disease progression.^9,15,16,17^

Some of the features of PAD and exercise-induced adaptations such as angiogenesis and vascular regeneration can be recapitulated in mouse models, which therefore can be utilized via transgenic, pharmacological, and gene transfer interventions to examine the molecular interaction between exercise and vascular regeneration. In rodent exercise models and various hindlimb ischemia models, key signaling modules have been identified in skeletal muscle to act as exercise sensors (eg, AMPK, ERRs) and drive paracrine angiogenesis. Similarly, exercise-generated metabolites from the skeletal muscle can act as paracrine activators of angiogenesis and regeneration (eg, NAD precursors, hydrogen sulfide, and lactate).^92,93,94,95^

Metabolite profiling in exercised and ischemic muscle may identify potential new mediators that can drive muscle angiogenesis or vascularization. Similarly, metabolite profiling of blood can identify exercise-activated circulatory factors that could be endocrine drivers of vascular regeneration. Combined use of single cell transcriptomics and tissue-specific transgenics for high probability genes could determine not only the cell types but also cell-specific genes that are critical for driving exercise-mediated vascular regeneration.

The links between exercise, immune cells, and stem cells also need research consideration. For example, tissue resident stem cells (eg, satellite cells) or immune cells (eg, macrophages), as well as bone marrow derived cells (eg, endothelial progenitor cells, neutrophils, and mesenchymal stem cells) could be activated by physical activity and contribute to exercise-induced vascular regeneration.

Finally, muscle-specific cargo that includes regulators beyond growth factors such as non-coding regulatory RNA or metabolites can be secreted in response to exercise via extracellular vesicles.^96,97^ How exercise regulates packaging/formation and release of such extracellular vesicles from the skeletal muscle is a relevant area of research for vascular regeneration. Overall, identification of core signaling pathways that mediate the effects of exercise on paracrine angiogenesis, immune/circulatory cell recruitment, and local stem cell activation could lead to “exercise-mimicking therapeutics” for vascular regeneration in cardiovascular disease.

## Key Points

1. Exercise has positive effects on cardiovascular health and vascular regeneration.
2. Peripheral arterial disease and skeletal muscle ischemia are excellent examples, where mechanisms of exercise promote vascular regeneration.
3. Nuclear receptors, transcriptional regulators, and kinases such as AMPK are exercise-mediators of muscle angiogenesis and revascularization.
4. While primarily working through the skeletal muscle, exercise regulators could also promote vascular regeneration by mobilizing muscle and endothelial stem cells and immune cells such as macrophages.
5. There remains a potential for developing exercise-mimicking therapeutics for vascular regeneration.
